# Fabrication of p-Type ZnO:N Films by Oxidizing Zn_3_N_2_ Films in Oxygen Plasma at Low Temperature

**DOI:** 10.3390/ma10030236

**Published:** 2017-02-27

**Authors:** Yuping Jin, Nuannuan Zhang, Bin Zhang

**Affiliations:** Institute of Modern Physics, Fudan University, Shanghai 200433, China; ypjin15@fudan.edu.cn (Y.J.); 15210200004@fudan.edu.cn (N.Z.)

**Keywords:** p-type ZnO:N films, oxygen vacancy (V_O_), zinc nitrite (Zn_3_N_2_), oxygen plasma

## Abstract

The oxygen vacancy (V_O_) is known as the main compensation center in p-type ZnO, which leads to the difficulty of fabricating high-quality p-type ZnO. To reduce the oxygen vacancies, we oxidized Zn_3_N_2_ films in oxygen plasma and successfully prepared p-type ZnO:N films at temperatures ranging from room temperature to 300 °C. The films were characterized by X-ray diffraction (XRD), non-Rutherford backscattering (non-RBS) spectroscopy, X-ray photoelectron spectroscopy, photoluminescence spectrum, and Hall Effect. The results show that the nitrogen atoms successfully substitute the oxygen sites in the ZnO:N films. The film prepared at room temperature exhibits the highest hole concentration of 6.22 × 10^18^ cm^−3^, and the lowest resistivity of 39.47 Ω∙cm. In all ZnO:N films, the V_O_ defects are reduced significantly. At 200 °C, the film holds the lowest value of V_O_ defects and the strongest UV emission. These results imply that oxygen plasma is very efficient in reducing V_O_ defects in p-type ZnO:N films, and could greatly reduce the reaction temperature. This method is significant for the development of ZnO-based optoelectronic devices.

## 1. Introduction

The broad band gap (3.37 eV at room temperature), the large exciton binding energy (60 meV at room temperature), the amenability to wet chemical etching, the realization of high quality single crystal, the possibility of low-temperature growth, and the transparent nature make ZnO a great potential material in optoelectronic devices—especially in the area of invisible and flexible electronics [1,2,3]. During the past few decades, flexible and transparent electronics have been an area of active research by several companies and by the scientific community [4,5]. With this technology, the fabrication of a wide range of innovative products will be possible, from flexible displays to wearable electronics. The most commonly used substrate materials are polyethylene terephthalate (PET) and polycarbonate (PC) because of their superior optical properties. As these plastic substrates are very temperature sensitive, the suitability for low-temperature deposition is very important. So far, ZnO could be fabricated at low temperature, and n-type ZnO growth is very mature. However, the difficulty of preparing reproducible and low-resistivity p-type ZnO has prevented ZnO from further development. The difficulty can arise from a variety of causes. Undoped ZnO shows intrinsic n-type conductivity, due to the probable impurity H [6,7]. While the intrinsic defects V_O_ (oxygen vacancies) and Zn_i_ (interstitial zinc atoms) may not cause n-type conductivity, they will compensate the acceptor dopants during the doping process [8]. Furthermore, the acceptor dopants generally have a low solubility in ZnO [9]. It has been believed that the most promising dopant for p-type ZnO is N, because of its similar radius to O. As N is not very soluble in ZnO, several groups have attempted p-type doping of ZnO by thermally oxidizing Zn_3_N_2_ [10,11,12]. The results show that a high concentration of N can be doped into ZnO through this method, and that the highest hole concentration can reach a level of 10^18^ cm^−3^. Nevertheless, the obtained p-type ZnO films contain a high concentration of V_O_, resulting in poor electrical property. In addition, the reported p-type ZnO:N films prepared by thermally oxidizing Zn_3_N_2_ films require very high temperature, which makes it impossible in flexible electronics. Therefore, the reduction of V_O_ concentration is obviously significant in p-type ZnO preparation. At the same time, searching for a low-temperature preparation method is quite meaningful.

In the photoluminescence (PL) spectra of ZnO, the broad-band emission locating in the visible region is commonly attributed to the V_O_-related defects [13,14]. To reduce the intensity of this peak, thermal annealing is usually adopted [15,16,17]. The mainly used annealing environment includes vacuum, oxygen, and nitrogen. Although the concentration of V_O_ can be reduced to a certain extent, the required annealing temperatures are very high.

In this letter, we report a simple method to fabricate p-type ZnO:N films. By oxidizing Zn_3_N_2_ films in oxygen plasma, the p-type ZnO:N films were obtained at low temperature. As the energy of oxygen plasma is very high, the transformation from Zn_3_N_2_ phase to ZnO phase will happen at low temperature. Moreover, the high energy oxygen ions could access the V_O_ sites in ZnO during the oxidizing process. As a result, the V_O_ defects can be significantly reduced. We have investigated the optical property of ZnO:N films prepared at different temperatures. Through the room-temperature photoluminescence spectra, we discovered that the oxygen plasma is quite efficient in reducing V_O_ defects in the ZnO:N films. Significantly, the hole concentration of film oxidized at room temperature reaches 6.22 × 10^18^ cm**^−^**^3^, almost greater than the reported results that were achieved with a high temperature process.

## 2. Materials and Methods

### 2.1. Zn_3_N_2_ Films Preparation

Zn_3_N_2_ samples were grown on quartz substrates by RF (radio frequency) reactive magnetron sputtering. A Zinc disk (99.999%), Ar gas (99.999%), and N_2_ gas (99.999%) were used. When the background pressure was less than 6 ×10^−4^ Pa, the Ar and N_2_ were introduced into the chamber, both with the flow rate of 30 standard-state cubic centimeters per minute (SCCM). The sputtering power, pressure, sputtering time, and substrate temperature were 40 W, 5 Pa, 30 min, and 200 °C, respectively. Before sputtering, a 15 min pre-sputtering was performed to clean the surface of the zinc disk. Here we have prepared two kinds of Zn_3_N_2_ films; the difference between them is the substrate temperature—one at 200 °C and the other at room temperature. Then, the two kinds of Zn_3_N_2_ films were cut into small pieces for the next oxidizing process.

### 2.2. Oxidizing Process

The Zn_3_N_2_ films were oxidized in a PECVD (plasma enhanced chemical vapor deposition) system. The background vacuum was better than 2 × 10^−5^ mbar. The O_2_ gas (99.999%) was inlet with the flow rate 9.5 SCCM. The working pressure and RF power were 400 mtorr and 100 W, respectively. The temperatures during oxidation were varied from room temperature (RT) to 400 °C. The oxidation time was set to 2 h. After oxidation, the samples were naturally cooled down to RT in the vacuum.

### 2.3. Measurements

X-ray diffraction (XRD) was performed to characterize the structural properties of the films on an X’Pert PRO diffractometer system with Cu Kα radiation (λ = 1.54060 Å). The composition and thickness of the films were measured by non-Rutherford backscattering (non-RBS) at the NEC 9SDH-2 3 MV pelletron tandem accelerator at Fudan University (details can be found in our former report [18]). The content and local chemical states of N were investigated by high-resolution X-ray photoelectron spectroscopy (XPS) at room temperature. The binding energy scale was calibrated using the C 1s line at 284.8 eV. Hall-effect measurement was carried out on an ACCENT HL5500PC Hall system using a Van der Pauw four-point configuration. Indium (In) electrodes were deposited on the four corners of the sample by RF magnetron sputtering at room temperature. The linear I–V behavior of all the samples indicated a good Ohmic contact between the In electrodes and the film layer. The photoluminescence (PL) property was measured with a He-Cd laser source with a wavelength of 325 nm at room temperature.

## 3. Results and Discussion

**Figure 1:**
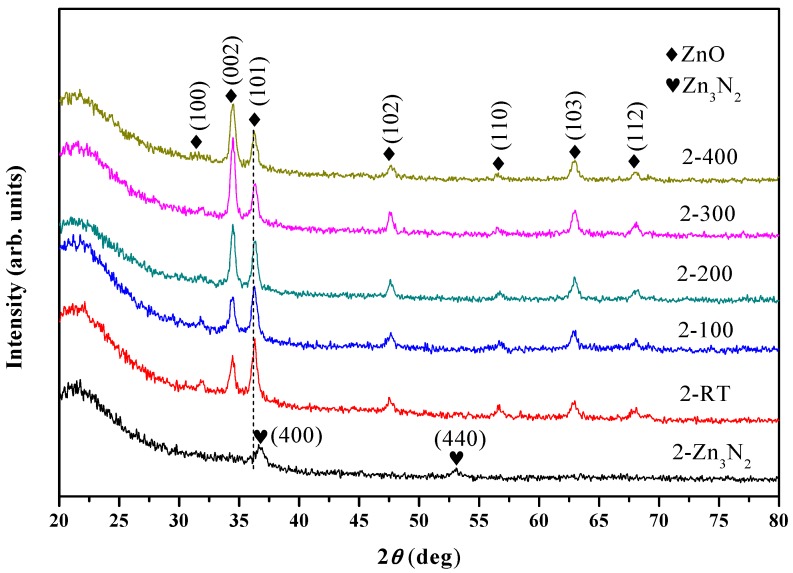
XRD patterns of as-grown 2-Zn_3_N_2_ film and ZnO:N films (labels 2-Zn_3_N_2_ and 2-RT/2-100/2-200/2-300/2-400 means that the Zn_3_N_2_ film was grown at 200 °C and that the oxidizing temperatures are room temperature (RT), 100 °C, 200 °C, 300 °C, and 400 °C, respectively).

Firstly, the as-grown Zn_3_N_2_ film grown at 200 °C and its oxidized films were investigated. The as-grown sample is labelled as 2-Zn_3_N_2_, and the oxidized films are labelled as 2-RT, 2-100, 2-200, 2-300, and 2-400, respectively. Figure 1 shows the XRD patterns of the Zn_3_N_2_ film labelled as 2-Zn_3_N_2_ and ZnO:N films. The Zn_3_N_2_ film shows two broad peaks at 36.76° and 52.98°, respectively, corresponding to (400) and (440) planes of Zn_3_N_2_ (JCPDS file 35-0762). After being oxidized in oxygen plasma, the film labelled as 2-Zn_3_N_2_ completely transformed into ZnO phase, even at RT. For the oxidized films, seven peaks emerge in the XRD patterns at 2θ = 31.80°, 34.44°, 36.30°, 47.60°, 56.63°, 62.86°, and 68.06°, which are attributed to (100), (002), (101), (102), (110), (103), and (112) planes of hexagonal wurtzite ZnO (JCPDS file 36-1451), respectively. The intensity of the ZnO (101) peak was the strongest at RT and 100 °C; it then weakened as the temperature increased. On the contrary, the intensity of ZnO (002) peak increased with the temperature and got stronger than that of the ZnO (101) peak at 200 °C. In other words, the prepared ZnO films tended to a (101) preferred orientation at RT and 100 °C, while to a (002) preferred orientation at temperatures higher than 100 °C. The growth of *c*-axis-oriented ZnO films was explained in terms of the low surface free energy for the (001) plane [19]. This is commonly found at high temperature. Nguyen et al. has reported that if the deposition is performed at non-equilibrium conditions (such as low temperature), another orientation can also be achieved [20]. Hence, low temperature may be responsible for the (101) preferred orientation of the 2-RT and 2-100 samples.

**Figure 2:**
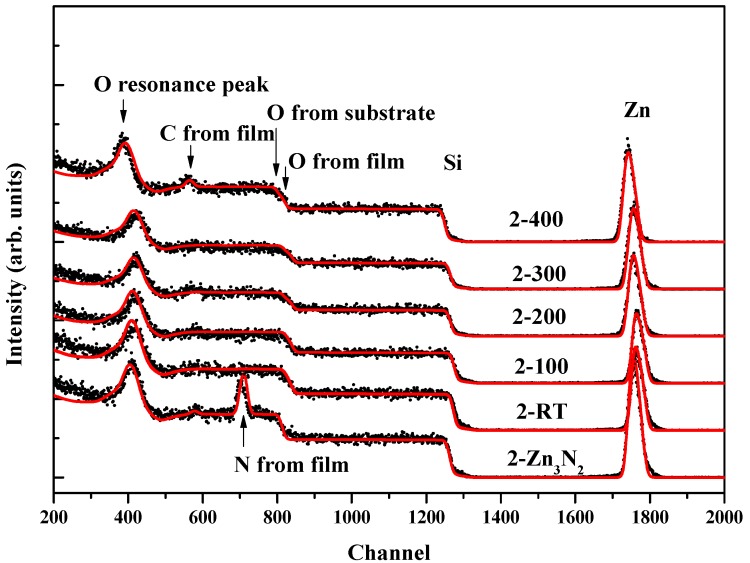
Non-Rutherford backscattering (non-RBS) spectra of as-grown 2-Zn_3_N_2_ film and ZnO:N films.

Figure 2 shows the non-RBS spectra of the films. By simulating the data with SIMNRA 6.0 code, we obtain the thickness and the components of the films. For all the films, the thicknesses are about 120 nm. The Zn_3_N_2_ film labelled as 2-Zn_3_N_2_ shows Zn/N ratio of 3:2, while the oxidized films all exhibit no N peak, with Zn/O ratio of 1:1. This means that the Zn_3_N_2_ and ZnO films were prepared successfully, and that the oxidation by oxygen plasma could totally change the Zn_3_N_2_ film into ZnO film, even at RT. This is the same with XRD results. In addition, we can see that the carbon contamination is inevitable in the samples because of the ex-situ oxidation process, which was demonstrated in our former report [18].

**Figure 3:**
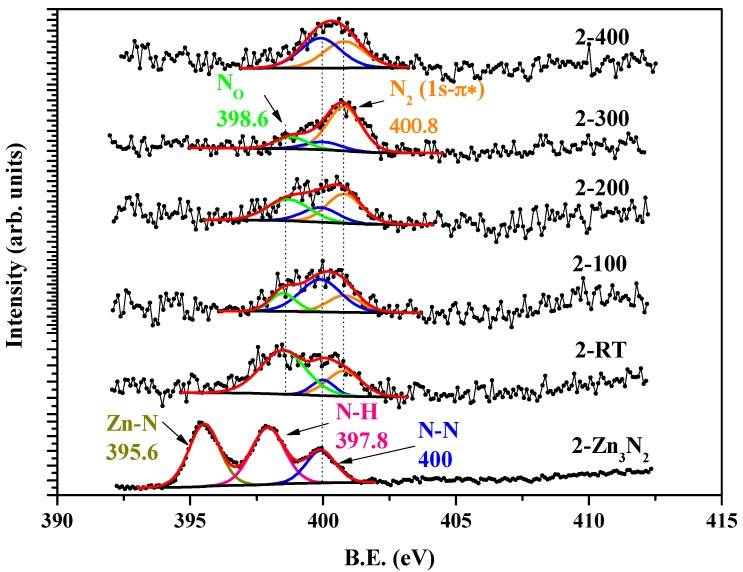
N 1s core-level X-ray photoelectron spectroscopy (XPS) spectra of as-grown 2-Zn_3_N_2_ film and ZnO:N films.

The XPS measurement was conducted to investigate the local chemical states of N atoms in the as-grown Zn_3_N_2_ film labelled as 2-Zn_3_N_2_ and ZnO:N, as shown in Figure 3. Three peaks were found in the as-grown Zn_3_N_2_ film at 395.6 eV, 397.8 eV, and 400 eV, which were assigned to Zn-N [21], N-H, and N-N bonds [22], respectively. This agrees with our previous results [18]. After being oxidized in the oxygen plasma, the Zn-N and N-H peaks disappeared, meaning that the film completely transformed into ZnO, and a new peak at 398.6 eV emerged at temperatures ranging from RT to 300 °C. This new peak is suggested to be N_O_ (substitutional nitrogen in oxygen site in the ZnO crystalline) [12,23,24], an acceptor in N-doped ZnO film. Another new peak at 400.8 eV was associated with the characteristic N 1s→π* transition in molecular nitrogen [22]. This indicates that the molecular nitrogen is produced during the oxidizing process. The contents of N and N_O_ of the ZnO:N films are listed in Table 1, which are obtained by simulating XPS data with Gaussian curves. At RT, the film contained 0.4 at % of N elements, among which 0.22 at % of N elements were existing as N_O_. As the temperature increased—except for the film oxidized at 100 °C—the total N contents in the ZnO:N films changed slightly, while the N_O_ contents reduced and vanished at 400 °C. At 100 °C, the film had 0.23 at % of total N content—the lowest among the ZnO:N films.

**Figure 4:**
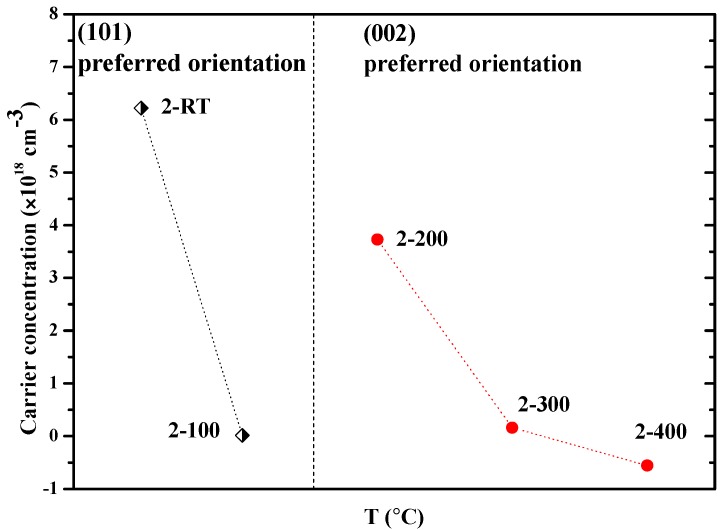
Carrier concentration for the ZnO:N films as a function of oxidizing temperature (T). Samples are classified by their crystal preferential orientation.

The Hall-effect measurements were conducted to obtain the electricity property of the films, as shown in Table 1. Claflin et al. mentioned that the low hole mobility makes ZnO:N susceptible to mixed-conduction effects. Even weak light exposure can potentially make a significant impact on the results [25]. Here, the Hall-effect measurements were conducted in the dark at room temperature. The magnetic field was 0.35 T. The as-grown Zn_3_N_2_ film labelled as 2-Zn_3_N_2_ exhibited n-type conduction, with carrier concentration of 1.08 × 10^19^ cm^−3^. After oxidizing in the oxygen plasma, the conduction type turned into p-type at temperatures ranging from RT to 300 °C, then into n-type again at 400 °C. The processing window of the p-type ZnO:N films was much lower than that by annealing at elevated temperatures (usually higher than 400 °C) in oxygen ambient. This could be attributed to the high energy of oxygen plasma. At RT, the energy of oxygen plasma was high enough to oxidize Zn_3_N_2_ into ZnO. When the temperature rose to 400 °C, the oxygen plasma was able to push N_O_ out of O site in the ZnO crystalline. At 100 °C, the film exhibited the lowest hole concentration. To ensure that the results are correct, we repeated the experiment. The results show that the hole concentration of the sample labelled as 2-100 was 2.23 × 10^16^ cm^−3^, almost the same as the results listed in Table 1. At RT, the film exhibited the highest hole concentration of 6.22 × 10^18^ cm^−3^, due to the highest N_O_ concentration. As the oxidizing temperature increased, the change of the hole concentration was the same as the change of N_O_ concentration. This means that the N_O_ is the dominant factor in the mechanism of N-doped p-type ZnO film using this method. This is because the concentration of the oxygen vacancies (V_O_)—acting as the compensate center in the p-type doping ZnO—in all the ZnO:N films were reduced efficiently. This will be discussed in detail in the part of the PL results. The carrier concentration for the ZnO:N films as a function of oxidizing temperature (T) is shown in Figure 4. As we discussed in the XRD results, the ZnO:N films preferred (101) plane orientation at RT and 100 °C, and (002) plane orientation at 200 °C, 300 °C, and 400 °C. Different crystal preferential orientation might affect the results of Hall-effect. Thus, the samples were clarified into two regions by their crystal preferential orientation. In the (101) preferred orientation region, the carrier concentration reduced from 6.22 × 10^18^ cm^−3^ to 1.67 × 10^16^ cm^−3^ as the temperature increased from RT to 100 °C. In the (002) preferred orientation region, the carrier concentration reached the highest value of 3.73 × 10^18^ cm^−3^ at 200 °C; it initially decreased as the temperature increased, then was followed by the transformation of conduction type from p-type to n-type at 400 °C. 

**Figure 5:**
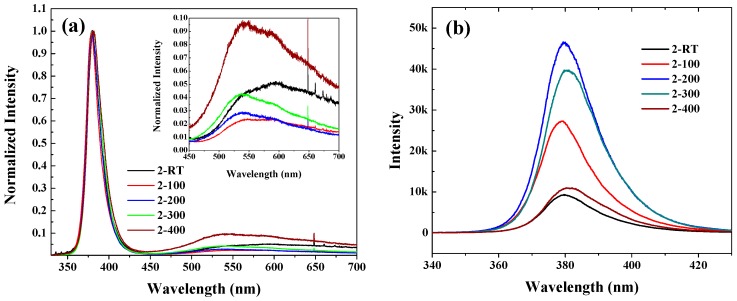
(**a**) Normalized photoluminescence spectra of ZnO:N films. The inset shows the enlarged photoluminescence spectra of ZnO:N films in the visual region; (**b**) Intensity of near-band emission peaks.

Figure 5 shows the photoluminescence spectra of the ZnO:N films. In Figure 5a, the spectra are normalized. The UV emissions of ZnO at 380 nm are quite strong to be found in all ZnO:N films. The weak broad peaks in the visible region from 450 to 700 nm are usually attributed to oxygen vacancy (V_O_) defects [13], acting as the donors in N-doped ZnO and compensating the effective N-acceptors. Compared with our previous work, where the Zn_3_N_2_ films were oxidized in the oxygen atmosphere, the V_O_ defects in all films were reduced obviously, meaning that the oxygen plasma has a high enough energy to access the V_O_ sites in the ZnO crystal. The enlarged figure of visible region (Figure 5a) shows the change of optical property with oxidizing temperature. The intensity of the V_O_ peak decreased initially and then increased when the oxidizing temperature was higher than 200 °C. On the contrary, the intensity of the UV emissions of ZnO at 380 nm (shown in Figure 5b) firstly increased and then decreased above 200 °C of the oxidizing temperature. That is, at 200 °C, the ZnO:N film maintained the strongest UV emission and the weakest deep level emission. In addition, high oxidizing temperature might increase the V_O_ defects, which generally happens at temperatures higher than 200 °C. In our experiments, above 200 °C, the V_O_ defects increased with the increasing oxidizing temperature.

**Figure 6:**
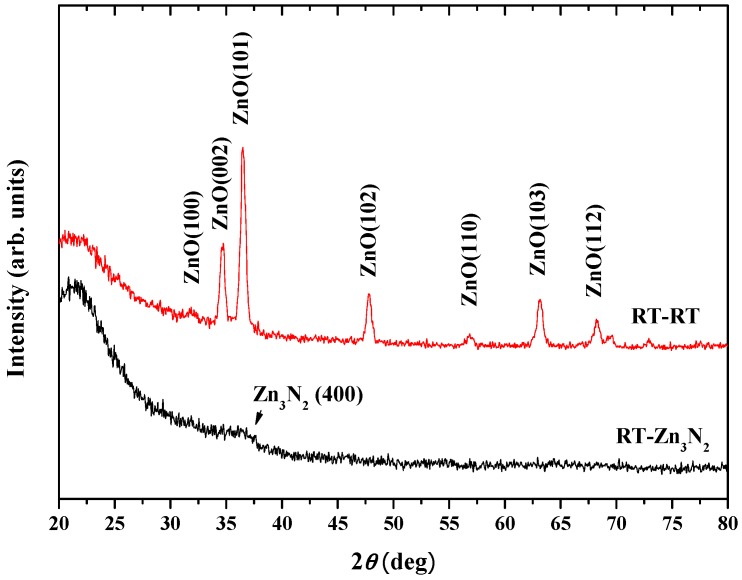
XRD patterns of Zn_3_N_2_ film grown at room temperature (labelled as RT-Zn_3_N_2_), and the ZnO:N film prepared by oxidizing RT-Zn_3_N_2_ at room temperature in the oxygen plasma atmosphere (labelled as RT-RT).

**Figure 7:**
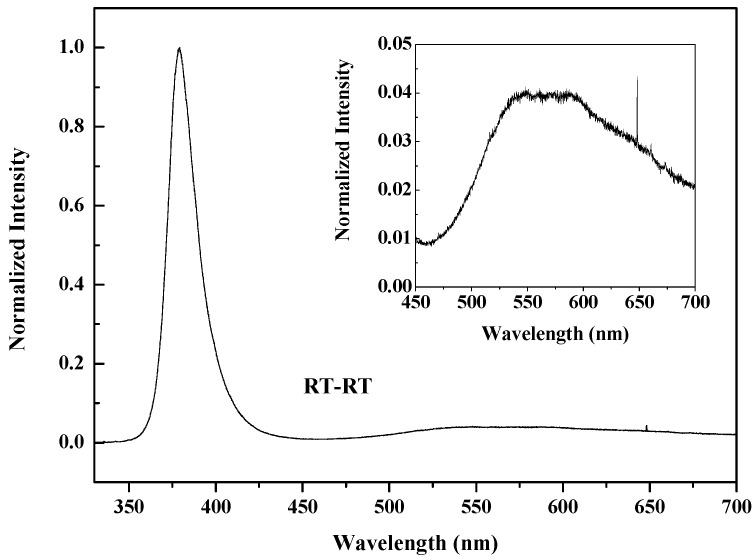
Normalized photoluminescence (PL) spectra of ZnO:N film prepared by oxidizing Zn_3_N_2_ film grown at RT. The insert shows the enlargement of the visible region.

As we mentioned above, the Zn_3_N_2_ film could be oxidized into p-type ZnO film even at RT, but the Zn_3_N_2_ films used above were prepared at 200 °C. In order to study the possibility of preparing p-type ZnO:N films at RT from beginning to end, the following experiment was performed. Using the same method, we prepared the Zn_3_N_2_ film at RT (labeled as RT-Zn_3_N_2_), then oxidized it in oxygen plasma atmosphere at RT (the oxidized sample is labeled as RT-RT). All the other experiment parameters were the same as before. The XRD patterns (Figure 6) show that the crystallinity of the Zn_3_N_2_ film deposited at RT (labelled as RT-Zn_3_N_2_) was not better than that of the Zn_3_N_2_ film deposited at 200 °C (labelled as 2-Zn_3_N_2_). However, after oxidizing in the oxygen plasma at RT, the Zn_3_N_2_ film deposited at RT also totally transformed into hexagonal wurtzite ZnO phase, with a (101) plane preferred orientation. From the photoluminescence spectra (Figure 7), we can also see the strong UV emission and the weak V_O_ defects peak. The electricity property of the RT-RT sample was also measured by Hall Effect, with resistivity of 7.67 × 10^4^ Ω∙cm and hole concentration of 1.81 × 10^16^ cm^−3^, which are not better than that of the 2-RT sample. E. Kaminska et al. suggested that a number of factors need to be considered in optimizing the technological procedure to obtain a high quality p-type ZnO [26]. This includes the microstructure of Zn_3_N_2_ and its potential contamination with hydrogen, the crystalline structure of the substrate, and the temperature and time of thermal processing. It was also mentioned that the microstructure of Zn_3_N_2_ was sensitive to the partial pressure of the nitrogen gas during deposition. In our experiments, the only difference between the two samples RT-RT and 2-RT was the deposited temperature of Zn_3_N_2_—one was at room temperature, another was at 200 °C. Obviously, the deposited temperature affects the microstructure of Zn_3_N_2._ To get good microstructure of Zn_3_N_2_ at room temperature, according to above suggestion, changing the partial pressure of the nitrogen gas during deposition might be an effective choice in further experiments. Therefore, oxidizing Zn_3_N_2_ with oxygen plasma is an efficient method to reduce the V_O_ in N-doped ZnO films. In addition, the preparation of p-type ZnO:N film could be realized at low temperature, even at RT.

## 4. Conclusions

In this letter, we report a simple method to prepare p-type ZnO:N films. By oxidizing Zn_3_N_2_ films in oxygen plasma atmosphere, the p-type N doped ZnO films were obtained at low temperature. The results show that:

(1) p-type N-doped ZnO films can be prepared by oxidizing Zn_3_N_2_ films in oxygen plasma atmosphere. Even at room temperature, the Zn_3_N_2_ film completely transformed into hexagonal wurtzite ZnO, with a hole concentration of 6.22 × 10^18^ cm^−3^ and a resistivity of 39.47 Ω∙cm.
(2) The Zn_3_N_2_ film prepared at room temperature could also be oxidized into p-type ZnO film in oxygen plasma atmosphere, but with a poor electrical property.
(3) The contents of N_O_ in the ZnO:N films decreased with the increasing oxidizing temperature, but exhibited the lowest value at 100 °C.
(4) The oxidizing process in oxygen plasma could significantly reduce the V_O_ defects in the ZnO:N films. The film obtained at 200 °C had the lowest value of V_O_ defects and the strongest UV emission.
(5) The oxidizing temperature did not always act as a positive factor in reducing the V_O_ defects in the preparation of ZnO:N films. When the temperature was higher than 200 °C, the concentration of V_O_ defects increased with the increasing temperature.

## Figures and Tables

**Table 1:** The electricity property of the as-grown 2-Zn_3_N_2_ and oxidized ZnO:N films.

Samples	N (at %)	N_O_ (at %)	Resistivity (Ω∙cm)	Hall Mobility (cm^2^·V^−1^·s^−1^)	Carrier Concentration (cm^−3^)	Type
2-Zn_3_N_2_	--	--	1.56 × 10^−2^	37.00	−1.08 × 10^19^	n
2-RT	0.40	0.22	39.47	0.03	6.22 × 10^18^	p
2-100	0.23	0.04	1.59 × 10^3^	0.24	1.67 × 10^16^	p
2-200	0.39	0.14	96.92	0.02	3.73 × 10^18^	p
2-300	0.43	0.07	2.57 × 10^2^	0.15	1.60 × 10^17^	p
2-400	0.43	--	26.89	0.42	−5.55 × 10^17^	n
